# In-Depth Characterization of Plant Growth Promotion Potentials of Selected Alkanes-Degrading Plant Growth-Promoting Bacterial Isolates

**DOI:** 10.3389/fmicb.2022.863702

**Published:** 2022-03-29

**Authors:** Fahad Alotaibi, Marc St-Arnaud, Mohamed Hijri

**Affiliations:** ^1^Institut de Recherche en Biologie Végétale, Département de Sciences Biologiques, Université de Montréal, Montréal, QC, Canada; ^2^Department of Soil Science, King Saud University, Riyadh, Saudi Arabia; ^3^African Genome Center, Mohammed VI Polytechnic University (UM6P), Ben Guerir, Morocco

**Keywords:** PGPR, alkanes, rhizoremediation, plant growth promotion, bioinoculants, 1-aminocyclopropane-1-carboxylate deaminase

## Abstract

The use of plant growth-promoting rhizobacteria (PGPR) as a bioremediation enhancer in plant-assisted phytoremediation requires several steps, consisting of the screening, selection, and characterization of isolates. A subset of 50 bacterial isolates representing a wide phylogenetic range were selected from 438 morphologically different bacteria that were originally isolated from a petroleum hydrocarbon (PHC)-polluted site of a former petrochemical plant. Selected candidate bacteria were screened using six conventional plant growth-promoting (PGP) traits, complemented with the genetic characterization of genes involved in alkane degradation, as well as other pertinent functions. Finally, the bacterial isolates were subjected to plant growth promotion tests using a gnotobiotic approach under normal and stressed conditions. Our results indicated that 35 bacterial isolates (70%) possessed at least four PGP traits. Twenty-nine isolates (58%) were able to utilize *n*-hexadecane as a sole carbon source, whereas 43 isolates (86%) were able to utilize diesel as the sole carbon source. The presence of catabolic genes related to hydrocarbon degradation was assessed using endpoint PCR, with the alkane monooxygenase (*alkB*) gene found in 34 isolates, the cytochrome P450 hydroxylase (*CYP153*) gene found in 24 isolates, and the naphthalene dioxygenase (*nah1*) gene found to be present in 33 isolates. Thirty-six strains (72%) promoted canola root elongation in the growth pouch assay. After several rounds of screening, seven bacterial candidates (individually or combined in a consortium) were tested for canola root and shoot growth promotion in substrates amended by different concentrations of *n*-hexadecane (0%, 1%, 2%, and 3%) under gnotobiotic conditions. Our results showed that *Nocardia* sp. (WB46), *Pseudomonas plecoglossicida* (ET27), *Stenotrophomonas pavanii* (EB31), and *Gordonia amicalis* (WT12) significantly increased the root length of canola grown in 3% *n*-hexadecane compared with the control treatment, whereas *Nocardia* sp. (WB46) and *Bacillus megaterium* (WT10) significantly increased shoot length compared to control treatment at the same concentration of *n*-hexadecane. The consortium had a significant enhancement effect on root length compared to all isolates inoculated individually or to the control. This study demonstrates that the combination of PGPR traits and the PHC degradation potential of bacteria can result in an enhanced beneficial effect in phytoremediation management, which could lead to the development of innovative bacterial inoculants for plants to remediate PHC-contaminated soils.

## Introduction

Human activities related to the petroleum and gas industry, such as exploration, extraction, refining, storage, and shipping, are polluting soil and water environments with petroleum hydrocarbons (PHCs; Alotaibi et al., 2021a). Aliphatic hydrocarbons (alkanes) are saturated hydrocarbons, representing the main constituents of crude oil, and are major soil contaminants (Chénier et al., 2003; Stroud et al., 2007). Alkanes are major soil pollutants, characterized by low chemical activity, low water solubility, and higher activation energies (Labinger and Bercaw, 2002; Rojo, 2009). Hexadecane (C_16_H_34_) is present in the aliphatic fraction of crude oil and is a main component of diesel fuel (Chénier et al., 2003). Hexadecane has been used as a model compound to study alkane biodegradation because of its presence in many diesel-contaminated soils and its well-characterized biodegradability (Chénier et al., 2003; Tara et al., 2014; Shiri et al., 2015; Balseiro-Romero et al., 2017a; Garrido-Sanz et al., 2019). The presence of these compounds in the environment adversely affects plant, animal, and human health (Arslan et al., 2014). Thus, the remediation of alkane-contaminated environments is a primary goal in the field of environmental biotechnology.

The use of the usual physical and chemical methods to cope with PHC contamination have shown many limitations (Alotaibi et al., 2021a). These conventional approaches are very expensive, only work for specific organic compounds and do not often result in the complete degradation of the contaminants; in addition, they are not environmentally friendly as they contribute to greenhouse gas emissions (Kuiper et al., 2004; Pilon-Smits, 2005; Behera, 2014). On the other hand, a biological method such as phytoremediation that relies on the plant–microbe partnership is a promising strategy for the remediation of soils contaminated with aliphatic hydrocarbons. Phytoremediation requires less maintenance effort, minimizes site disturbances, and is a cost-effective and less destructive approach (Alotaibi et al., 2021a).

Plants, due to their exudates, metabolite diversity and enzymatic machinery, can adapt and alleviate stressful conditions such as the presence of hydrocarbons in soil (Pilon-Smits, 2005). However, plant growth and biomass production are often limited under such harsh conditions and subsequently phytoremediation efficiency is reduced and plant mortality is increased (Glick and Stearns, 2011). Plant growth-promoting rhizobacteria (PGPR) can be used to enhance plant growth in stressful conditions, thus enhancing phytoremediation efficiency (Tara et al., 2014; Balseiro-Romero et al., 2017a; Oleńska et al., 2020). PGPR can alleviate stress in plants and reduce the phytotoxicity of hydrocarbons *via* many mechanisms, such as reducing soil nutrient deficiencies (fixing nitrogen, solubilizing phosphorus, and enhancing iron uptake), synthesizing plant growth-promoting (PGP) hormones, suppressing ethylene production *via* 1-aminocyclopropane-1-carboxylate (ACC) deaminase (ACCD) activity (Schlaeppi and Bulgarelli, 2015; Backer et al., 2018; Oleńska et al., 2020), and by virtue of their pollutant-degrading pathways and metabolic activities (Arslan et al., 2014; Xu et al., 2018).

The development of a database and collection of bacterial isolates characterized for PGP traits and hexadecane degradation potential can assist in the selection of the most promising strains for further advancement as bioaugmentation inoculants in phytoremediation strategies for diesel-contaminated soils. In this study, 50 bacterial strains were selected from 438 morphologically different bacteria that were isolated from bulk and rhizosphere soils of plants growing in a petrochemical site contaminated with PHCs (Alotaibi et al., 2021b). The strains were selected based on the fact that they covered a wide phylogenetic affiliation range, their ability to possess various PGP activities and their wide-spectrum hydrocarbon degradation potential. We hypothesize that combined traits of PGP and hexadecane degradation potential occur in bacteria isolated from an aged petroleum-hydrocarbon polluted site. The specific objectives of this study were: to define and compare the PGP traits of selected bacterial strains qualitatively and quantitatively; to test their ability to utilize 1% (v/v) sterilized *n*-hexadecane and 1% (v/v) diesel as a sole carbon source; to screen for the presence of the stress tolerance gene (*acdS*) encoding ACCD and the dinitrogenase reductase gene (*nifH*) encoding (nitrogen fixation); to search for the presence of hydrocarbon-degrading genes (*alkB*, *CYP153*, and *nah*); and to assess their plant growth promotion potential under gnotobiotic conditions in growth pouches. The outcome of this study can be an effective approach for the developing integrated microbial inoculants for bioremediation biotechnology applications.

## Materials and Methods

### Bacterial Strains

The 50 bacterial strains used in this study are a subset of a larger collection of 438 morphologically different bacteria isolated from bulk soil and the rhizosphere of *Salix purpurea* and *Eleocharis obtusa* plants growing in a site highly polluted with petroleum-hydrocarbons (Alotaibi et al., 2021b). This site is in Varennes, Quebec, Canada (45°43 N, 73°22 W), with an allocated area of approximately 5,000 m^2^. These bacterial strains were selected based on their phylogenetic affiliations to cover major bacterial lineages in order to increase taxonomic, genetic and functional diversities. The identification of bacterial strains was performed using Sanger sequencing of the 16S rRNA gene, as described in Alotaibi et al. (2021b). The isolation source and the species affiliations of bacterial strains used in this study are summarized in Table 1. The bacterial strains were stored at −80°C.

**Table 1:** List of bacterial isolates used in this study.

Isolation code	Environmental niche	Isolation medium^a^	Phylum/Family	NCBI taxonomic identity (accession number)
ST4	Bulk soil	1/10TSA	Actinobacteria/Nocardiaceae	*Rhodococcus ruber* (MZ430450)
ST15	Bulk soil	1/10TSA	Gammaproteobacteria/Pseudomonadaceae	*Pseudomonas* sp. (MZ430461)
ST25	Bulk soil	1/10TSA	Gammaproteobacteria/Xanthomonadaceae	*Stenotrophomonas nitritireducens* (MZ430471)
ST45	Bulk soil	1/10TSA	Actinobacteria/Gordoniaceae	*Gordonia amicalis* (MZ430491)
SB26	Bulk soil	B–H_amended diesel	Actinobacteria/Rhodobacteraceae	*Paracoccus* sp. (MZ430412)
SB32	Bulk soil	B–H_amended diesel	Actinobacteria/Microbacteriaceae	*Microbacterium hatanonis* (MZ430418)
SB36	Bulk soil	B–H_amended diesel	Gammaproteobacteria/Moraxellaceae	*Acinetobacter pittii* (MZ430422)
SB38	Bulk soil	B–H_amended diesel	Gammaproteobacteria/Pseudomonadaceae	*Pseudomonas stutzeri* (MZ430424)
SB39	Bulk soil	B–H_amended diesel	Actinobacteria/Microbacteriaceae	*Microbacterium oxydans* (MZ430425)
SB41	Bulk soil	B–H_amended diesel	Gammaproteobacteria/Moraxellaceae	*Acinetobacter* sp. (MZ430427)
SB45	Bulk soil	B–H_amended diesel	Gammaproteobacteria/Pseudomonadaceae	*Pseudomonas mosselii* (MZ430431)
SB49	Bulk soil	B–H_amended diesel	Betaproteobacteria/Oxalobacteraceae	*Massilia oculi* (MZ430435)
SB50	Bulk soil	B–H_amended diesel	Alphaproteobacteria/Sphingomonadaceae	*Sphingobium yanoikuyae* (MZ430436)
ET5	*Eleocharis* rhizosphere	1/10TSA	Actinobacteria/Microbacteriaceae	*Microbacterium testaceum* (MZ430211)
ET10	*Eleocharis* rhizosphere	1/10TSA	Alphaproteobacteria/Rhizobiaceae	*Rhizobium selenitireducens* (MZ430216)
ET25	*Eleocharis* rhizosphere	1/10TSA	Firmicutes/Bacillaceae	*Bacillus marisflavi* (MZ430231)
ET27	*Eleocharis* rhizosphere	1/10TSA	Gammaproteobacteria/Pseudomonadaceae	*Pseudomonas plecoglossicida* (MZ430233)
ET33	*Eleocharis* rhizosphere	1/10TSA	Betaproteobacteria/Comamonadaceae	*Delftia lacustris* (MZ430239)
ET46	*Eleocharis* rhizosphere	1/10TSA	Gammaproteobacteria/Yersiniaceae	*Serratia* sp. (MZ430252)
ET49	*Eleocharis* rhizosphere	1/10TSA	Gammaproteobacteria/Enterobacteriaceae	*Enterobacter bugandensis* (MZ430255)
EB6	*Eleocharis* rhizosphere	B–H_amended diesel	Betaproteobacteria/Burkholderiaceae	*Chitinimonas taiwanensis* (MZ430152)
EB26	*Eleocharis* rhizosphere	B–H_amended diesel	Gammaproteobacteria/Aeromonadaceae	*Aeromonas hydrophila* (MZ430172)
EB31	*Eleocharis* rhizosphere	B–H_amended diesel	Gammaproteobacteria/Xanthomonadaceae	*Stenotrophomonas pavanii* (MZ430177)
EB35	*Eleocharis* rhizosphere	B–H_amended diesel	Betaproteobacteria/Comamonadaceae	*Comamonas odontotermitis* (MZ430181)
EB37	*Eleocharis* rhizosphere	B–H_amended diesel	Actinobacteria/Microbacteriaceae	*Lysinimonas* sp. (MZ430183)
EB43	*Eleocharis* rhizosphere	B–H_amended diesel	Gammaproteobacteria/Pseudomonadaceae	*Pseudomonas entomophila* (MZ430189)
WT8	*Salix* rhizosphere	1/10TSA	Actinobacteria/Streptomycetaceae	*Streptomyces atriruber* (MZ430334)
WT10	*Salix* rhizosphere	1/10TSA	Firmicutes/Bacillaceae	*Bacillus megaterium* (MZ430336)
WT12	*Salix* rhizosphere	1/10TSA	Actinobacteria/Gordoniaceae	*Gordonia amicalis* (MZ430338)
WT17	*Salix* rhizosphere	1/10TSA	Gammaproteobacteria/Pseudomonadaceae	*Pseudomonas kilonensis* (MZ430343)
WT19	*Salix* rhizosphere	1/10TSA	Actinobacteria/Micrococcaceae	*Pseudarthrobacter siccitolerans* (MZ430345)
WT34	*Salix* rhizosphere	1/10TSA	Actinobacteria/Micrococcaceae	*Arthrobacter* sp. (MZ430360)
WT39	*Salix* rhizosphere	1/10TSA	Actinobacteria/Streptomycetaceae	*Streptomyces atratus* (MZ430365)
WB17	*Salix* rhizosphere	B–H_amended diesel	Actinobacteria/Micrococcaceae	*Paenarthrobacter nitroguajacolicus* (MZ430283)
WB23	*Salix* rhizosphere	B–H_amended diesel	Betaproteobacteria/Comamonadaceae	*Variovorax paradoxus* (MZ430289)
WB25	*Salix* rhizosphere	B–H_amended diesel	Alphaproteobacteria/Sphingomonadaceae	*Sphingomonas sanxanigenens* (MZ430291)
WB31	*Salix* rhizosphere	B–H_amended diesel	Gammaproteobacteria/Pseudomonadaceae	*Pseudomonas frederiksbergensis* (MZ430297)
WB40	*Salix* rhizosphere	B–H_amended diesel	Actinobacteria/Micrococcaceae	*Pseudarthrobacter siccitolerans* (MZ430306)
WB46	*Salix* rhizosphere	B–H_amended diesel	Actinobacteria/Nocardiaceae	*Nocardia* sp. (MZ430312)
WB48	*Salix* rhizosphere	B–H_amended diesel	Actinobacteria/Nocardiaceae	*Streptomyces umbrinus* (MZ430314)
WB49	*Salix* rhizosphere	B–H_amended diesel	Actinobacteria/Nocardioidaceae	*Nocardioides alpinus* (MZ430315)
WB51	*Salix* rhizosphere	B–H_amended diesel	Actinobacteria/Gordoniaceae	*Nocardia asteroides* (MZ430317)
WB54	*Salix* rhizosphere	B–H_amended diesel	Actinobacteria/Intrasporangiaceae	*Phycicoccus bigeumensis* (MZ430320)
SA7	Bulk soil	ACCD	Gammaproteobacteria/Enterobacteriaceae	*Pantoea agglomerans* (MZ430101)
EA5	*Eleocharis* rhizosphere	ACCD	Gammaproteobacteria/Enterobacteriaceae	*Klebsiella oxytoca* (MZ430073)
EA9	*Eleocharis* rhizosphere	ACCD	Gammaproteobacteria/Enterobacteriaceae	*Enterobacter cancerogenus* (MZ430077)
EA21	*Eleocharis* rhizosphere	ACCD	Actinobacteria/Microbacteriaceae	*Curtobacterium* sp. (MZ430089)
WA8	*Salix* rhizosphere	ACCD	Gammaproteobacteria/Erwiniaceae	*Raoultella terrigena* (MZ430128)
WA19	*Salix* rhizosphere	ACCD	Gammaproteobacteria/Enterobacteriaceae	*Citrobacter* sp. (MZ430139)
WA25	*Salix* rhizosphere	ACCD	Gammaproteobacteria/Pseudomonadaceae	*Pseudomonas thivervalensis* (MZ430145)

a *Isolation media abbreviations: B–H_amended diesel, Bushnell–Haas medium amended with 1% diesel; TSA, Trypticase Soy Agar; and ACCD, 1-aminocyclopropane-1-carboxylate (ACC) deaminase enrichment culture method*.

Stock cultures were preserved in 20% glycerol at −80°C. When reviving bacteria, isolates were cultured in 50 ml of 1/2 strength Trypticase Soy Broth (TSB) (Difco Laboratories Inc. Detroit, Michigan, United States) at room temperature for 48 h with continuous agitation at 150 rpm on a gyratory shaker.

### Screening for *in vitro* PGP Characteristics

#### Phosphate Solubilization

The inorganic phosphate solubilization activity of bacterial isolates was determined using both qualitative and quantitative assays as described in Nautiyal (1999). In the qualitative assay using solid agar plates, fresh pure bacterial isolates were grown in half-strength TSB at 28°C for 48 h with continuous agitation at 150 rpm in a rotary shaker. Then, 10 μl of growing bacterial culture was spot-inoculated into the center of NBRIP (the National Botanical Institute’s phosphate growth medium) agar plates containing tri-calcium phosphate as the sole inorganic phosphate source (Nautiyal, 1999). The NBRIP agar plates were incubated at 28°C for 14 days and a clear zone around inoculated colonies indicated the solubilization of inorganic phosphate. The test was replicated three times.

In the quantitative liquid assay, a loopful of pure bacterial isolates growing on 1/10 Trypticase Soy Agar (TSA) plates were inoculated into 125-ml Erlenmeyer flasks containing 50 ml freshly sterilized liquid NBRIP medium supplemented with tri-calcium phosphate as the sole inorganic phosphate source. The cultures were grown at 28°C under continuous agitation at 150 rpm in a rotary shaker for up to 14 days. Five-milliliter aliquots were centrifuged at 10,000 g for 10 min, and the supernatant was filtered through a 0.2-μm Millipore filter and used for soluble P determination using ammonium molybdate reagent (Fiske and Subbarow, 1925). The blue-color compound was measured by reading the absorbance at 650 nm using a multimode microplate spectrophometer against a standard curve KH_2_PO_4_. The test was replicated three times.

#### Indole-3-Acetic Acid Production

The production of IAA by bacterial isolates was determined using both qualitative and quantitative assays as described in Patten and Glick (2002). Bacterial isolates were first cultured overnight in 5 ml of DF salts minimal medium, and then 20 μl aliquots were transferred into 15-ml Falcon tubes containing 5 ml of DF salts minimal medium supplemented with tryptophan (1 mg ml^−1^) as auxin precursor. Cultures were grown in a shaker (120 rpm) for 48 h at 28°C. One-milliliter aliquots of bacterial cultures were then centrifuged at 9,500 g for 2 min and 100 μl of supernatant were added to a 96-well plate, followed by the addition of 100 μl of Salkowski’s reagent, and the 96-well plate was incubated in the dark for 30 min at room temperature. Bacterial isolates producing IAA were characterized by the formation of a distinct red color. To quantify IAA produced by bacterial isolates, the absorbance was measured at 535 nm using a multimode microplate spectrophotometer against a standard curve of commercial IAA (Sigma-Aldrich, United States). The test was replicated three times.

#### Siderophore Syntheses

Siderophore production by bacterial isolates was determined qualitatively using the Chrome-Azurol S (CAS) assay as described in Alexander and Zuberer (1991). Pure bacterial isolates were grown in half-strength TSB at 28°C for 48 h with continuous agitation at 150 rpm in a rotary shaker, and 10 μl of the growing bacterial culture was spot-inoculated into the centers of CAS-agar plates. The CAS-agar plates were incubated at 28°C for 72 h, and bacterial isolates showing an orange halo were considered positive for siderophore synthesis (Schwyn and Neilands, 1987). The test was replicated three times.

Siderophore production quantification was estimated using the CAS-Shuttle assay performed in high-throughput mode using a 96-well format, as described in Payne (1994). Briefly, bacterial strains were inoculated into an iron-deficient MM9 medium to induce siderophore production and grown at 28°C under continuous agitation. After 48 h, 100 μl of cell-free supernatant was mixed with 100 μl of CAS dye and 2 μl of shuttle solution. The 96-well plate was incubated in the dark for 15 min, and the absorbance was measured at 630 nm using a multimode microplate spectrophotometer. The test was replicated three times.

#### Ammonia Production

The ammonia production by bacterial isolates was evaluated in both qualitative and quantitative assays as described in Cappuccino and Sherman (1992) and as outlined in Dutta et al. (2015). The qualitative estimation of ammonia production was carried out by inoculating fresh bacterial isolates into 10-ml test tubes of peptone water (peptone 10 g. L^−1^; NaCl 5 g. L^−1^; 1 L dH_2_O), and bacterial cultures were incubated for 72 h at 28°C. Then, 1 ml aliquots of bacterial culture were transferred to 2 ml tubes and 50 μl of Nessler’s reagent [10% HgI^2^; 7% KI; 50% aqueous solution of NaOH (32%)] were added to each tube. A color change of the mix to yellow indicates ammonia production, with a weak yellow indicating of small amount of production and a deep yellow being a sign of the maximum capacity of ammonia production (Marques et al., 2010). To quantify ammonia production by bacterial isolates, the absorbance was measured at 450 nm against a standard curve of ammonium sulfate using a multimode microplate spectrophotometer. The test was replicated three times.

#### ACCD Activity

ACCD activity was assessed by monitoring the bacterial isolate’s ability to grow on DF minimal salts medium containing ACC as a sole nitrogen source (Penrose and Glick, 2003). Pure bacterial isolates were grown in half-strength TSB at 28°C for 48 h under continuous agitation at 150 rpm in a rotary shaker. A loopful of each bacterial isolate growing in liquid culture was streaked into a DF minimal salts agar plate containing 3 mM ACC solution, which was spread into the agar plate immediately prior to use. Plates were incubated at 28°C for up to 1 week. The presence of growth in the DF-ACC agar plates was considered positive. Bacterial strains were classified using a rating scale as follows: −, no growth; +, slightly growth; ++, moderate growth; and +++, heavy growth. The test was replicated three times.

ACCD activity was also confirmed *via* PCR amplification of the *acdS* gene (Blaha et al., 2006). More details regarding procedure and PCR conditions are given below.

#### Nitrogen Fixation

Bacterial isolates were evaluated regarding their capacity to grow on an N-deficient combined carbon medium (Rennie, 1981). Bacterial cultures were grown in half-strength TSB at 28°C for 48 h under continuous agitation at 150 rpm in a rotary shaker. A loopful of each bacterial isolate growing in liquid culture was streaked into the N-deficient combined carbon medium agar plate and incubated at 28°C for up to 1 week. The presence of growth in the agar plates was considered positive. Bacterial strains were classified using a rating scale as follows: −, no growth; +, slightly growth; ++, moderate growth; and +++, heavy growth. The test was replicated three times.

Nitrogen fixation activity was also confirmed using PCR amplification of the *nifH* gene (Rösch et al., 2002). More details regarding the procedure and PCR conditions are given below.

### Catabolic Gene Detection Using PCR Amplifications

PCR analysis was used to assess the presence of hydrocarbon-degrading genes and PGPR genes in bacterial isolates selected in this study. Primers used to detect the presence of genes and PCR conditions are presented in Supplementary Table S1.

PCR reactions for the analysis of the *alkB* gene were performed in a reaction volume of 25 μl, which consisted of 1× PCR Buffer (Qiagen, Toronto, Canada), 0.8 μM of each primer, 0.2 mM of dNTP mix, 0.5 mM of MgCl_2_, 0.2 mg ml^−1^ of BSA (Amersham Biosciences, Mississauga, Canada), 1.25 U of *Taq* DNA polymerase (Qiagen, Toronto, Canada), and 1 μl purified genomic DNA (Kloos et al., 2006). For the detection of the *CYP153* gene, PCR reactions were prepared as for the *alkB* gene (Wang et al., 2010). In addition, PCR *nah* gene detection was conducted as previously described in Baldwin et al. (2003). Briefly, PCR analyses were performed in a reaction volume of 25 μl, which consisted of 1× PCR buffer, 0.5 μM of each primer, 1 μl of dNTP (Qiagen, Toronto, Canada), 0.5 mM of MgCl_2_, 0.2 mg ml^−1^ of BSA, 1 U of *Taq* DNA polymerase, and 1 μl purified genomic DNA. For the detection of the *acdS* gene, PCR reactions were prepared as for the *nah* gene (Blaha et al., 2006). Finally, for the detection of the *nifH* gene, PCRs were performed in a reaction volume of 25 μl of 1× PCR buffer, 0.5 μM of each primer, 0.5 μl of dNTP, 0.5 μl of MgCl_2_, 0.2 mg ml^−1^ of BSA, 1 U of *Taq* DNA polymerase, and 1 μl purified genomic DNA (Rösch et al., 2002).

The presence and length of PCR products were verified by electrophoresis with GelRed-stained 1.5% agarose gels using the Gel-Doc system (Bio-Rad Laboratories, Mississauga, Canada).

### PGP Potential of Bacterial Isolates Under Gnotobiotic Conditions

#### Inoculum Preparation

Bacterial isolates (Table 1) were first grown in fresh 1/10 TSA plates and incubated for 72–96 h at 28°C. Then, pure colonies of each isolate were inoculated separately into a 500 ml Erlenmeyer flask containing 250 ml half-strength TSB medium. Bacterial isolates were incubated on a rotary shaker (150 rpm) at 28°C for 48 h (except for the following isolates, which were grown for up to 120 h at 28°C: *Rhodococcus ruber* ST4, *Sphingobium yanoikuyae* SB50, *Lysinimonas* sp. EB37, *Gordonia amicalis* WT12, *Sphingomonas* sp. WB25, *Nocardioides alpinus* WB49, *Gordonia* sp. WB51, and *Phycicoccus* sp. WB54). The optical density (OD) of bacterial cells was measured and adjusted to an OD_600_ value of 1. Bacterial suspensions were harvested *via* centrifugation (15 min at 5,000 g), washed three times in phosphate buffer saline (PBS; Difco Laboratories, Detroit, United States), and resuspended in 20 ml sterile tap water. Serial dilutions were then prepared in PBS and spread on 1/10 TSA plates, and incubated at 28°C for 72 h. This yielded cell densities of approximately 10^9^ (colony-forming units) cfu ml^−1^.

#### Seed Inoculation

Canola (cv. 4,187 RR) seeds were surface-sterilized by washing with ethanol (95% v.v^−1^) for 30 s, followed by soaking in NaCIO (2.5% v.v^−1^) for 10 min under constant gentle shaking (Hynes et al., 2008). Seeds were rinsed with sterile distilled water 10 times in order to remove excess sodium hypochlorite. The seeds were then air-dried by placing them in a biosafety cabinet for 24 h. Sub-samples of surface-sterilized seeds were picked randomly and placed onto 1/10-strength TSA plates and incubated at 28°C for 24 h to further check for any contamination. Surface-sterilized seeds were soaked in 5 ml of bacterial suspension for 4 h with gentle shaking in a rotary shaker to allow the bacteria to penetrate into the seeds. For the control treatment (without bacterial inoculum), seeds were soaked in 5 ml autoclaved distilled H_2_O.

#### Root Elongation Assay

The root elongation assays were conducted under gnotobiotic conditions using growth pouches as previously described in Lifshitz et al. (1987). Seed growth pouches (16.5 cm × 18 cm) containing chromatographic filter paper (Mega International, Minneapolis, United States) were filled with 10 ml of sterile half-strength N-free Hoagland’s nutrient solution, wrapped in aluminum foil and sterilized at 121°C for 20 min prior to seeding. Ten seeds soaked in the bacterial suspension were aseptically sown inside the growth pouches and five replicate pouches were used for each treatment and for the control. After seed germination, pouches were thinned to five seeds per pouch. The pouches were wrapped with Saran plastic wrap to minimize the loss of moisture and covered with aluminum foil to prevent light from reaching plant roots. The moisture content was kept constant during the time course of the experiment *via* additions of sterile distilled water and half-strength N-free Hoagland nutrient solution, on an alternative day’s basis, using aseptic techniques. The seeds were grown in growth pouches for 7 days at 28°C, with a 16/8 h day/night cycle, before the root measurements were taken.

### Growth of Bacterial Isolates in MSM With (1%) *n*-Hexadecane and (1%) Diesel

Bacterial isolates were tested for their ability to utilize either 1% (v/v) filter-sterilized *n*-hexadecane or 1% (v/v) diesel in mineral salt medium (MSM) by measuring the cell density at 600 nm. Bacterial isolates were first grown in half-strength TSB at 28°C for 48 h with continuous agitation at 150 rpm. Then, cells were collected *via* centrifugation, washed three times with PBS, and resuspended in sterile dH_2_O, and 10 μl was used to inoculate MSM amended with 1% *n*-hexadecane or 1% diesel. The assay was carried out in 125 ml Erlenmeyer flasks containing 50 ml sterile MSM and the *n*-hexadecane or diesel as carbon source, incubated at 28°C under continuous agitation at 150 rpm on a rotary shaker. After a week, cells growth was measured at 600 nm and compared with a control containing no carbon source. The experiment was carried out in triplicate.

### Gnotobiotic Assay Under Alkane Stress Conditions

Seven candidates that performed well in different assays were selected and tested gnotobiotically for their plant growth promotion potential under different concentrations of *n*-hexadecane (0%, 1%, 2%, and 3%).

The bacterial strains tested in this experiment were *Acinetobacter* sp. strain SB41, *Pseudomonas putida* strain ET27, *Stenotrophomonas maltophilia* strain EB31, *Bacillus megaterium* strain WT10, *G. amicalis* strain WT12, *Arthrobacter* sp. strain WT19, and *Nocardia* sp. strain WB46. The bacterial inoculum was prepared as described above.

The experiment was performed utilizing growth pouches as described above, with modifications. The ability of bacterial strains to enhance plant growth under alkane stress conditions was tested by including different concentrations of *n*-hexadecane (0%, 1%, 2%, and 3%) in the sterile half-strength N-free Hoagland nutrient solution in the growth pouches. Briefly, 10 seeds soaked in the bacterial suspension were aseptically sown inside the growth pouches and five replicated pouches were used for each treatment and control. After seed germination, pouches were thinned to five seeds per pouch. The pouches were wrapped with Saran plastic wrap to minimize the loss of moisture and covered with aluminum foil to prevent light from reaching the plant roots. The seeds were grown in growth pouches for 7 days at 28°C, with a 16/8 h day/night cycle, before the root and shoot measurements were made.

#### Growth of Bacterial Isolates on Different Concentrations of *n*-Hexadecane

The growth of bacterial strains was measured in sterile 50 ml MSM medium containing different concentrations of *n*-hexadecane (1%, 2%, and 3%) as the carbon source in 125 ml Erlenmeyer flasks. Bacterial cultures were grown as described in above and then the bacterial cultures were incubated at 28°C under continuous agitation at 150 rpm on a rotary shaker. Bacterial growth was determined by measuring the cell density at 600 nm every day for up to 7 days. Non-inoculated control treatments were included at each concentration. The experiment was carried out in triplicate.

### Statistical Analyses

The growth pouch study was carried out in a completely randomized design. In the first growth pouch study, the data were presented as means and SDs, and the difference between treatments compared to the control were analyzed using Dunnett’s test (*p* = 0.05). In the second growth pouch experiment, the differences between treatments were analyzed using one-way ANOVA at a 5% significance level with Tukey’s *post-hoc* test, using JMP software (SAS Institute Inc. Cary, NC, United States). A Venn diagram was generated using *InteractiVenn* software (Heberle et al., 2015).

## Results

### PGP Traits of Bacterial Isolates

A total of 50 bacterial isolates were screened for six different PGP traits and the presence of two genes encoding nitrogenase (*nifH*) and ACCD (*acdS*). The results of screening tests are shown in Table 2. Fourteen strains (28%) were able to solubilize calcium phosphate in the liquid medium (Table 2). Among these, several strains showed excellent P solubilization ability. *B. megaterium* strain WT10 showed the highest solubilization activity with 690.86 μg ml^−1^ calcium phosphate (Table 2), followed by *G. amicalis* strain WT12, *Curtobacterium* sp. strain EA21, and *Pseudomonas fluorescens* strain WT17, which were able to solubilize 567.12 μg ml^−1^, 525.4 μg ml^−1^, and 476.48 μg ml^−1^ calcium phosphate, respectively (Table 2).

**Table 2:** Screening of selected bacterial strains for PGP traits.

Strain code	Identity	IAA production^a^ (μg ml^−1^)	Phosphate solubilization (μg ml^−1^)	Siderophore production (%)	Ammonia production (μmol ml^−1^)	ACC deaminase^b^	Nitrogen fixation^c^	PCR^d^	
								*acdS*	*nifH*
ST4	*Rhodococcus ruber*	25.93	−	−	9.59	+	+	+	+
ST15	*Pseudomonas* sp.	11.51	−	13.4	7.35	++	+	−	+
ST25	*Stenotrophomonas nitritireducens*	21.48	−	7.6	9.81	+++	−	+	−
ST45	*Gordonia amicalis*	−	−	−	−	++	+++	+	+
SB26	*Paracoccus* sp.	−	−	−	9.47	−	−	−	−
SB32	*Microbacterium hatanonis*	−	−	9.16	7.39	++	+++	−	−
SB36	*Acinetobacter pittii*	−	420.36	22.3	−	+++	+	+	+
SB38	*Pseudomonas stutzeri*	13.78	−	32.1	8.11	−	−	+	−
SB39	*Microbacterium oxydans*	6.51	−	8.2	12.51	++	−	−	−
SB41	*Acinetobacter* sp.	4.39	369.28	−	7.25	+++	++	−	+
SB45	*Pseudomonas mosselii*	19.10	−	12.3	6.42	+++	+++	+	+
SB49	*Massilia oculi*	7.67	−	17.1	7.36	−	−	−	−
SB50	*Sphingobium yanoikuyae*	15.34	−	−	5.93	++	++	+	+
ET5	*Microbacterium testaceum*	11.01	−	−	8.23	−	−	−	−
ET10	*Rhizobium selenitireducens*	44.31	−	26.7	9.05	−	+++	−	+
ET25	*Bacillus marisflavi*	−	−	−	8.61	+++	−	−	−
ET27	*Pseudomonas plecoglossicida*	11.80	424.68	36.4	7.07	+++	+++	+	+
ET33	*Delftia lacustris*	5.05	−	−	−	+++	−	+	−
ET46	*Serratia* sp.	13.47	381.51	24.8	7.06	++	+	−	+
ET49	*Enterobacter bugandensis*	9.18	−	31.1	8.59	++	++	−	+
EB6	*Chitinimonas taiwanensis*	−	−	−	13.05	++	−	−	−
EB26	*Aeromonas hydrophila*	1.67	−	−	10.50	−	−	−	−
EB31	*Stenotrophomonas pavanii*	29.44	380.79	19.6	11.46	++	+	−	+
EB35	*Comamonas odontotermitis*	−	−	14.9	13.95	+	−	+	+
EB37	*Lysinimonas* sp.	−	−	−	8.64	−	−	−	−
EB43	*Pseudomonas*	0.45	−	−	8.89	++	++	−	+
WT8	*entomophila*	0.53	72.16	−	9.16	−	−	−	−
WT10	*Streptomyces atriruber Bacillus*	−	690.86	−	9.27	−	−	−	−
WT12	*megaterium Gordonia amicalis*	0.45	567.12	−	7.97	++	+++	+	+
WT17	*Pseudomonas kilonensis*	8.41	476.48	29. 3	6.41	+++	++	+	+
WT19	*Pseudarthrobacter siccitolerans*	11.56	−	10.1	6.70	−	+	−	+
WT34	*Arthrobacter* sp.	10.32	−	−	10.34	−	−	−	−
WT39	*Streptomyces atratus*	−	−	−	5.0	−	−	−	−
WB17	*Paenarthrobacter nitroguajacolicus*	−	−	12.1	6.86	+++	++	+	+
WB23	*Variovorax paradoxus*	−	−	−	−	++	+++	+	+
WB25	*Sphingomonas sanxanigenens*	1.14	−	8.3	9.10	++	−	−	−
WB31	*Pseudomonas frederiksbergensis*	9.68	449.86	22.8	7.19	+++	++	+	+
WB40	*Pseudarthrobacter siccitolerans*	7.88	−	−	7.28	−	−	−	−
WB46	*Nocardia* sp.	1.46	−	8.2	6.67	−	−	−	−
WB48	*Streptomyces umbrinus*	−	−	−	9.05	++	+++	+	−
WB49	*Nocardioides alpinus*	8.49	−	−	9.71	−	−	−	−
WB51	*Nocardia asteroides*	−	−	−	8.60	++	++	+	+
WB54	*Phycicoccus bigeumensis*	0.24	72.16	−	9.35	−	−	−	−
SA7	*Pantoea agglomerans*	19.37	−	29.1	8.42	+++	−	+	−
EA5	*Klebsiella oxytoca*	31.32	−	18.7	7.85	+++	+++	+	+
EA9	*Enterobacter cancerogenus*	10.58	−	36.4	8.25	+++	+	+	+
EA21	*Curtobacterium* sp.	44.13	525.4	−	8.60	+++	+++	−	+
WA8	*Raoultella terrigena*	13.54	−	−	7.97	+++	++	+	+
WA19	*Citrobacter* sp.	−	338.35	−	8.42	+++	+	+	+
WA25	*Pseudomonas thivervalensis*	−	324.68	19.3	7.11	+++	+++	+	+

*Values are means of three replicates ± SD*.

a *Indole-3-acetic acid*.

b *1-aminocyclopropane-1-carboxylate deaminase “−” means showed no growth on agar plates, “^+^” means showed low growth on agar plates, “^++^” means showed medium growth on agar plates, and “^+++^” means showed heavy growth on agar plates*.

c *The presence of growth in the agar plates was considered positive for nitrogen fixation, “−” means showed no growth on agar plates, “^+^” means showed low growth on agar plates, “^++^” means showed medium growth on agar plates, and“^+++^” means showed heavy growth on agar plates*.

d *“−” indicates the absence of PCR products and “^+^” indicates the presence of PCR products for the functional genes—*acdS*, ACCD gene and *nifH*, nitrogen fixation gene*.

Out of 50 bacterial strains, 34 strains (68%) were able to produce IAA after 48 h of incubation with a 1 mg ml^−1^ supplement of tryptophan as an auxin precursor (Table 2). Three bacterial strains showed the highest IAA production among all the strains, specifically *Rhizobium* sp. strain ET10, *Curtobacterium* sp. strain EA21, and *Klebsiella* sp. EA5, which produced 44.31 μg ml^−1^, 44.13 μg ml^−1^, and 31.32 μg ml^−1^ IAA, respectively (Table 2).

Twenty-four bacterial strains (48%) were able to synthesize siderophores (Table 2). The maximum production of siderophores by bacterial strains were observed in *Pseudomonas putida* strain ET27, *Enterobacter* sp. strain EA9, *Pseudomonas stutzeri* strain SB38, *Enterobacter cancerogenus* strain ET49, and *Pseudomonas fluorescens* strain WT17, which produced around 29% or above of siderophore units (Table 2).

Most of the bacterial strains under investigation were able to produce ammonia (Table 2). Most bacterial isolates produced ammonia in the range of 5.00 to 10.50 μmol ml^−1^ (Table 2). Four bacterial strains showed the maximum ammonia production among the strains, namely, *Comamonas* sp. strain EB35, *Chitinimonas* sp. strain EB6, *Microbacterium oxydans* strain SB39, and *S. maltophilia* strain EB31 (Table 2).

All bacterial isolates were further screened qualitatively for ACCD and N fixation, of which 34 isolates (68%) demonstrated ACCD activity and also showed the presence of an ACCD gene (*acdS*) (Table 2). Additionally, 28 isolates (56%) showed the ability to fix atmospheric N_2_ and the presence of the N fixation gene (*nifH*) (Table 2).

### Root Elongation Assay

**Figure 1:**
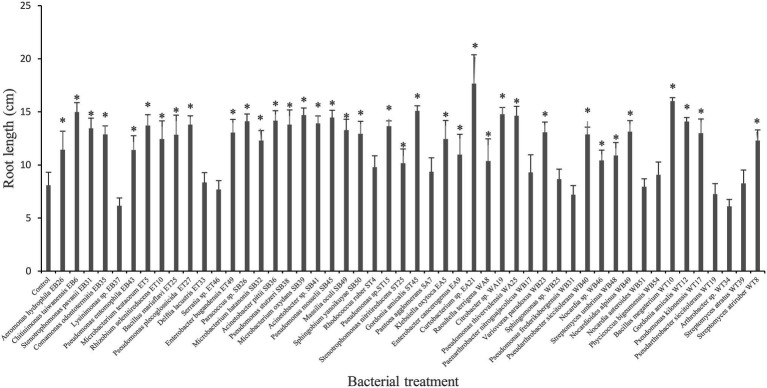
Effect of selected plant growth-promoting rhizobacteria (PGPR) bacterial isolates on root length (cm) of canola plants measured after 7 days of growth. Error bars represent SDs and * indicates a significant difference compared to the control according to a Dunnett test, *p* ≤ 0.05.

The bacterial isolates were tested on canola root elongation under gnotobiotic conditions. The results indicated that the highest canola root elongation effect was induced by the following bacterial isolates: *Curtobacterium* sp. EA21, *B. megaterium* WT10, and *Gordonia* sp. ST45, which significantly increased (*p* ≤ 0.05) canola root elongation by 118%, 98%, and 86%, respectively, compared with the control treatment (Figure 1). Other isolates that significantly increased (*p* ≤ 0.05) canola root length included *Citrobacter* sp. WA19, *Pseudomonas thivervalensis* WA25, *Microbacterium oxydans* SB39, *Pseudomonas mosselii* SB45, *Pseudomonas stutzeri* SB38, *Acinetobacter pittii* SB36, *Pseudomonas putida* ET27, *S. maltophilia* EB31, *G. amicalis* WT12, *Arthrobacter* sp. WB40, and *Klebsiella* sp. EA5 (Figure 1). In contrast, bacterial isolates *Arthrobacter* sp. WT34, *Pseudomonas* sp. WB31 and *Gordonia* sp. WB51 tended to inhibit canola root elongation, although this effect was not significant at *p* = 0.05 (Figure 1).

### *n*-Hexadecane and Diesel Degradation Potential, and Presence of *alkB*, *CYP153* and *nahA* Genes in Bacterial Isolates

*n*-hexadecane was used as the sole carbon source for the growth of 29 isolates (58%) (Table 3). Some isolates, such as *G. amicalis* ST45, *Comamonas odontotermitis* EB35, *Pseudomonas fluorescens* WT17, *Nocardia* sp. WB46, *Nocardia asteroides* WB51, and *Phycicoccus bigeumensis* WB54, showed high growth rates when they were cultivated in MSM medium supplemented with *n*-hexadecane as the sole source of carbon and energy (Table 3).

**Table 3:** Ability of bacterial isolates to grow on aliphatic compounds and to possess hydrocarbon degradation genes.

Strain	Identity^a^	Isolation medium	Growth in diesel^b^	Growth in hexadecane^c^	PCR^d^		
					*alkB*	*CYP153*	*nah*
ST4	*Rhodococcus ruber*	1/10TSA	+++	+++	+	+	+
ST15	*Pseudomonas* sp.	1/10TSA	+	−	−	−	+
ST25	*Stenotrophomonas nitritireducens*	1/10TSA	++	−	+	−	−
ST45	*Gordonia amicalis*	1/10TSA	++++	++++	+	+	−
SB26	*Paracoccus* sp.	B–H_amended diesel	+	−	−	+	+
SB32	*Microbacterium hatanonis*	B–H_amended diesel	++	−	+	−	+
SB36	*Acinetobacter pittii*	B–H_amended diesel	−	++	+	−	+
SB38	*Pseudomonas stutzeri*	B–H_amended diesel	++	+	−	+	+
SB39	*Microbacterium oxydans*	B–H_amended diesel	+	−	+	−	−
SB41	*Acinetobacter* sp.	B–H_amended diesel	+++	++	+	−	+
SB45	*Pseudomonas mosselii*	B–H_amended diesel	++	−	−	+	+
SB49	*Massilia oculi*	B–H_amended diesel	−	+	+	−	+
SB50	*Sphingobium yanoikuyae*	B–H_amended diesel	+++	+	+	−	+
ET5	*Microbacterium testaceum*	1/10TSA	+	−	−	−	+
ET10	*Rhizobium selenitireducens*	1/10TSA	+	−	+	+	+
ET25	*Bacillus marisflavi*	1/10TSA	++	−	−	−	+
ET27	*Pseudomonas plecoglossicida*	1/10TSA	+++	+	+	+	+
ET33	*Delftia lacustris*	1/10TSA	−	+	+	−	+
ET46	*Serratia* sp.	1/10TSA	−	+	+	−	+
ET49	*Enterobacter bugandensis*	1/10TSA	+++	−	+	+	+
EB6	*Chitinimonas taiwanensis*	B–H_amended diesel	++++	+	−	+	+
EB26	*Aeromonas hydrophila*	B–H_amended diesel	+	+	+	−	−
EB31	*Stenotrophomonas pavanii*	B–H_amended diesel	++++	+++	+	+	+
EB35	*Comamonas odontotermitis*	B–H_amended diesel	+	++++	+	−	+
EB37	*Lysinimonas* sp.	B–H_amended diesel	+	−	+	−	+
EB43	*Pseudomonas entomophila*	B–H_amended diesel	+	+	+	−	−
WT8	*Streptomyces atriruber*	1/10TSA	−	−	−	−	+
WT10	*Bacillus megaterium*	1/10TSA	+++	+	+	−	−
WT12	*Gordonia amicalis*	1/10TSA	++++	++++	+	+	+
WT17	*Pseudomonas kilonensis*	1/10TSA	+++	+	−	+	−
WT19	*Pseudarthrobacter siccitolerans*	1/10TSA	+	++	+	−	+
WT34	*Arthrobacter* sp.	1/10TSA	++	+	+	−	−
WT39	*Streptomyces atratus*	1/10TSA	+	−	−	−	−
WB17	*Paenarthrobacter nitroguajacolicus*	B–H_amended diesel	++++	+	+	+	+
WB23	*Variovorax paradoxus*	B–H_amended diesel	++	+	+	−	+
WB25	*Sphingomonas sanxanigenens*	B–H_amended diesel	+++	−	+	−	−
WB31	*Pseudomonas frederiksbergensis*	B–H_amended diesel	+	+++	−	+	−
WB40	*Pseudarthrobacter siccitolerans*	B–H_amended diesel	++	++	−	−	+
WB46	*Nocardia* sp.	B–H_amended diesel	++++	++++	+	−	+
WB48	*Streptomyces umbrinus*	B–H_amended diesel	++	+	+	+	−
WB49	*Nocardioides alpinus*	B–H_amended diesel	+	−	−	+	+
WB51	*Nocardia asteroides*	B–H_amended diesel	+++	++++	+	+	+
WB54	*Phycicoccus bigeumensis*	B–H_amended diesel	−	++++	+	−	−
SA7	*Pantoea agglomerans*	ACCD	+	−	+	−	−
EA5	*Klebsiella oxytoca*	ACCD	+	−	−	+	−
EA9	*Enterobacter cancerogenus*	ACCD	+++	+	+	+	+
EA21	*Curtobacterium* sp.	ACCD	+	−	−	+	−
WA8	*Raoultella terrigena*	ACCD	++	−	+	+	+
WA19	*Citrobacter* sp.	ACCD	−	+	−	+	−
WA25	*Pseudomonas thivervalensis*	ACCD	++	+	+	+	+

a *Indicates 16S rDNA identity of bacterial strains with their closest type strains in GenBank*.

b *Indicates growth capability of bacterial strains on 1% (v:v) diesel in MSM. ++++, +++, ++, +, and −, indicating the growth capability from strong to weak with diesel as a sole carbon and energy source, measured by optical density at 600 nm, after 1 week incubation at 28°C. ++++, growth (OD600 > 1); +++, growth (OD600 > 0.6); ++, growth (0.6 > OD600 > 0.2); +, growth (OD600 < 0.2); and −, no growth*.

c *Indicates growth capability of bacterial strains on 1% (v:v) *n*-hexadecane in MSM. ++++, +++, ++, +, and −, indicating the growth capability from strong to weak with *n*-hexadecane as a sole carbon and energy source, measured by optical density at 600 nm, after 1 week incubation at 28°C. ++++, growth (OD600 > 1); +++, growth (OD600 > 0.6); ++, growth (0.6 > OD600 > 0.2); +, growth (OD600 < 0.2); and −, no growth*.

d *Indicates “−” absence of PCR products and “^+^” presence of PCR products for the functional genes: *alkB*, alkane monooxygenase; *CYP153*, cytochrome P450 hydroxylase; and *nah*, naphthalene dioxygenase*.

Similarly, 43 bacterial isolates (86%) were able to utilize diesel as the sole carbon source (Table 3). Isolates *R. ruber* ST4, *G. amicalis* ST45, *Comamonas odontotermitis* EB35, *B. megaterium* WT10, *G. amicalis* WT12, *Pseudomonas kilonensis* WT17, *Paenarthrobacter nitroguajacolicus* WB17, *Sphingomonas sanxanigenens* WB25, *Nocardia* sp. WB46, *Nocardia asteroides* WB51, and *Enterobacter cancerogenus* EA9 showed the highest growth rate when they were cultivated in MSM medium supplemented with diesel as the sole source for carbon and energy (Table 3).

The detection of the presence of functional genes related to PHC-degradation—*alkB*, *CYP153*, and *nah1*—was used to evaluate the biodegradation ability of bacterial isolates. The *alkB* gene was detected in 34 isolates (68%) by PCR amplification (Table 3). The *CYP153* gene was also detected in 24 isolates (48%) (Table 3), whereas 33 bacterial isolates (66%) possessed the *nah1* gene (Table 3).

**Figure 2:**
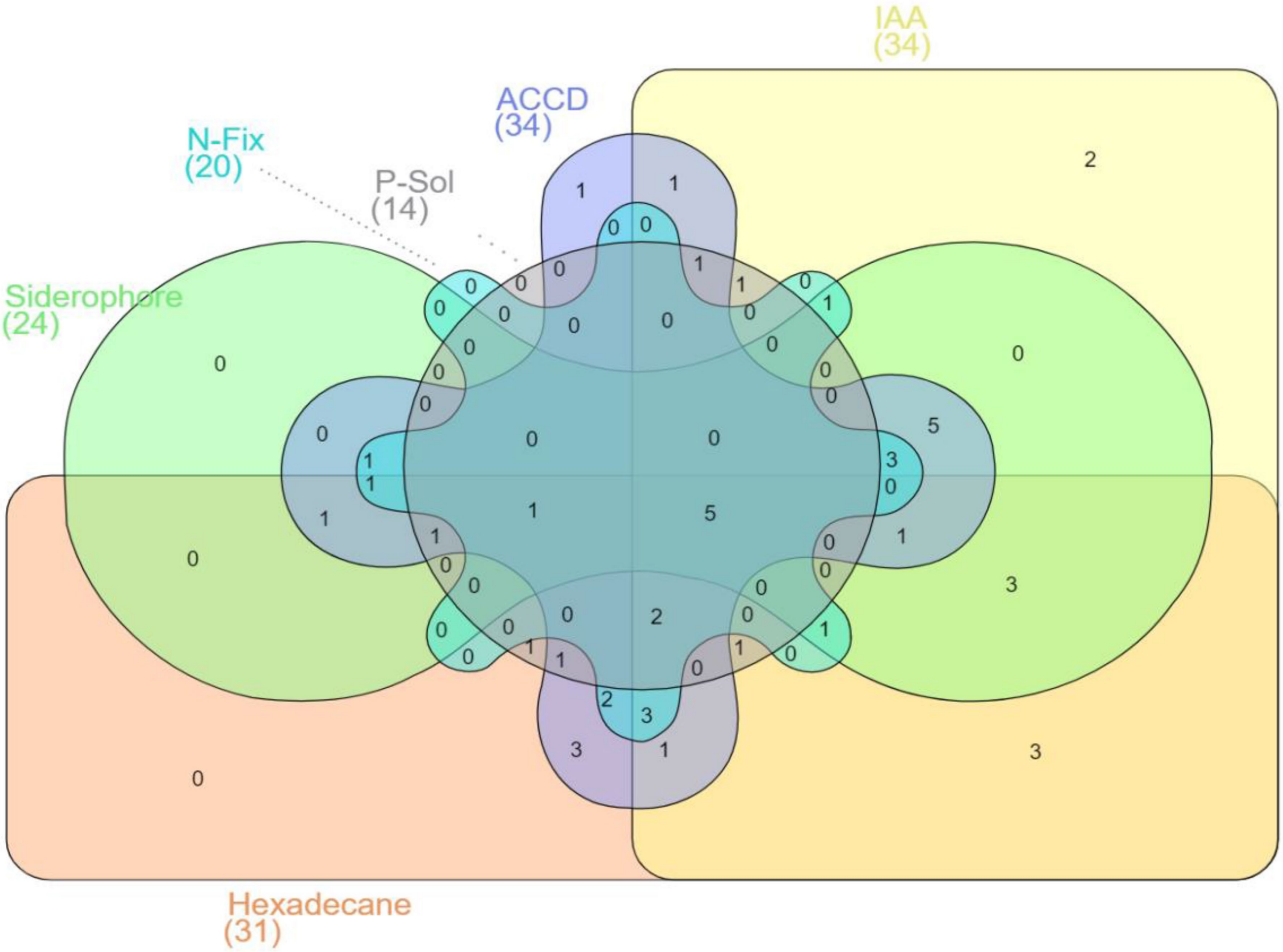
Venn diagram representation of 50 rhizobacterial strains, showing positive results for hexadecane degradation potential and different plant growth-promoting (PGP) traits (with five strains showing positive results for all the traits under investigation).

Notably, five hexadecane-degrading bacterial isolates possessed all PGP traits under investigation (Figure 2) specifically, *Pseudomonas putida* ET27, *Serratia* sp. ET46, *S. maltophilia* EB31, *Pseudomonas fluorescens* WT17, and *Pseudomonas* sp. WB31 (Figure 2).

### Canola Growth Promotion under *n*-Hexadecane Gradient

**Figure 3:**
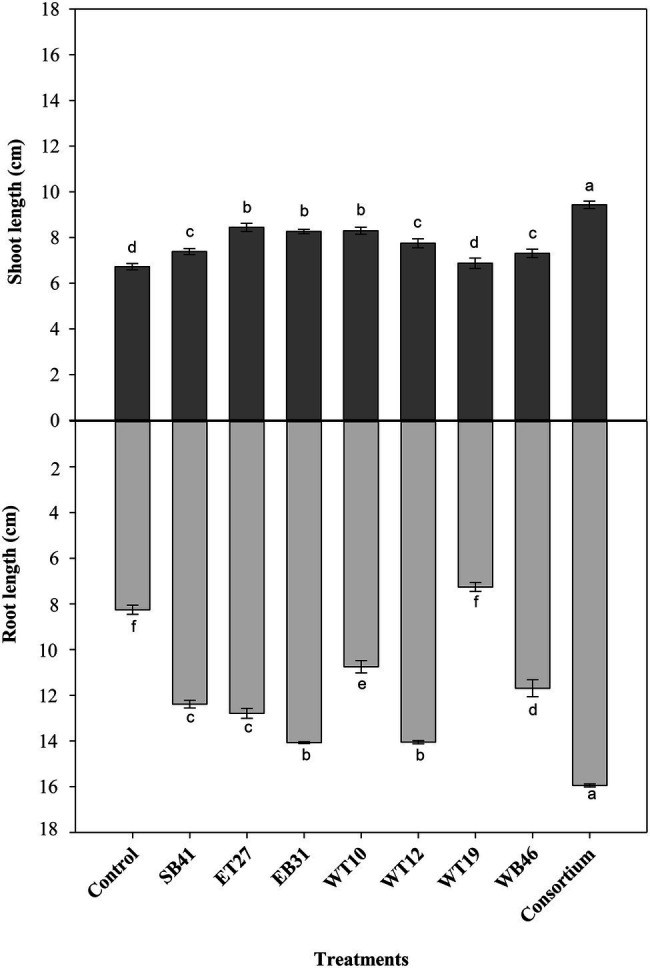
Effect of selected PGPR bacterial strains on root and shoot length (cm) of canola plants measured after 7 days of growth in the presence of 0% *n*-hexadecane. Error bars represent SDs, and different letters indicate significance according to Tukey’s *post-hoc* test at *p* ≤ 0.05.

Further screening tests were performed on bacterial isolates in order to assess their PGP activity using canola plants in growth pouches amended or not with a gradient of *n*-hexadecane concentrations ranging from 0% as a control to 3%. Under the conditions of no hydrocarbon stress (0% *n*-hexadecane), the consortium significantly increased (*p* ≤ 0.05) root length compared to all isolates inoculated individually and to the control (Figure 3). All bacterial isolates, except *Pseudarthrobacter siccitolerans* WT19, significantly increased (*p* ≤ 0.05) their root length compared to the control treatment (Figure 3). The highest growth promotion was observed in inoculations with *Stenotrophomonas pavanii* EB31 and *G. amicalis* WT12 (Figure 3). Similarly, all bacterial treatments significantly increased (*p* ≤ 0.05) the shoot lengths of canola plants compared to the control (Figure 3). The highest growth promotion was observed in the consortium treatment (Figure 3).

**Figure 4:**
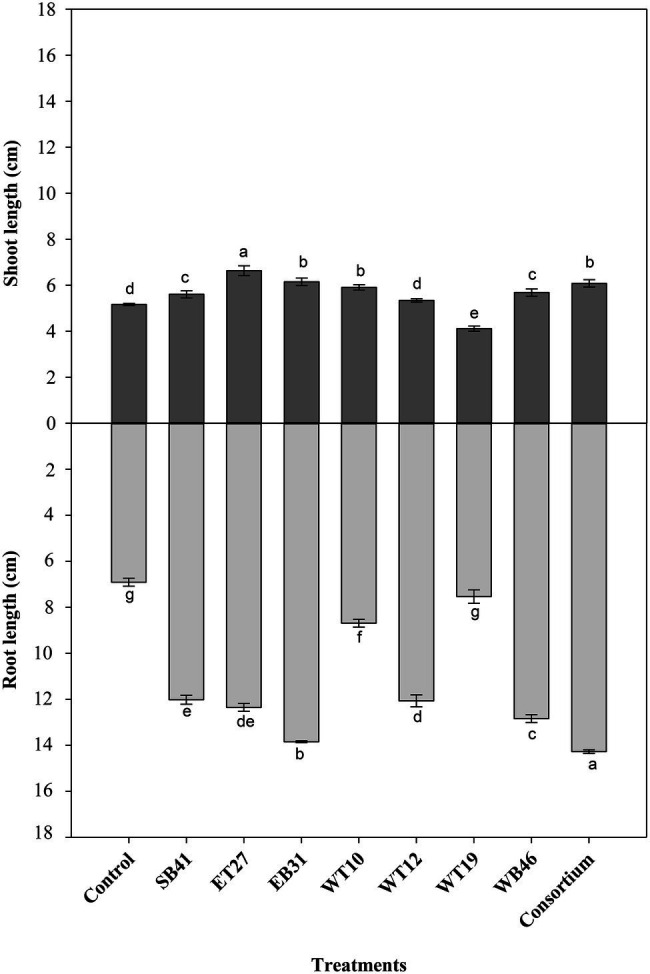
Effect of selected PGPR bacterial strains on root and shoot length (cm) of canola plants measured after 7 days of growth in the presence of 1% *n*-hexadecane. Error bars represent SDs, and different letters indicate significance according to Tukey’s *post-hoc* test at *p* ≤ 0.05.

**Figure 5:**
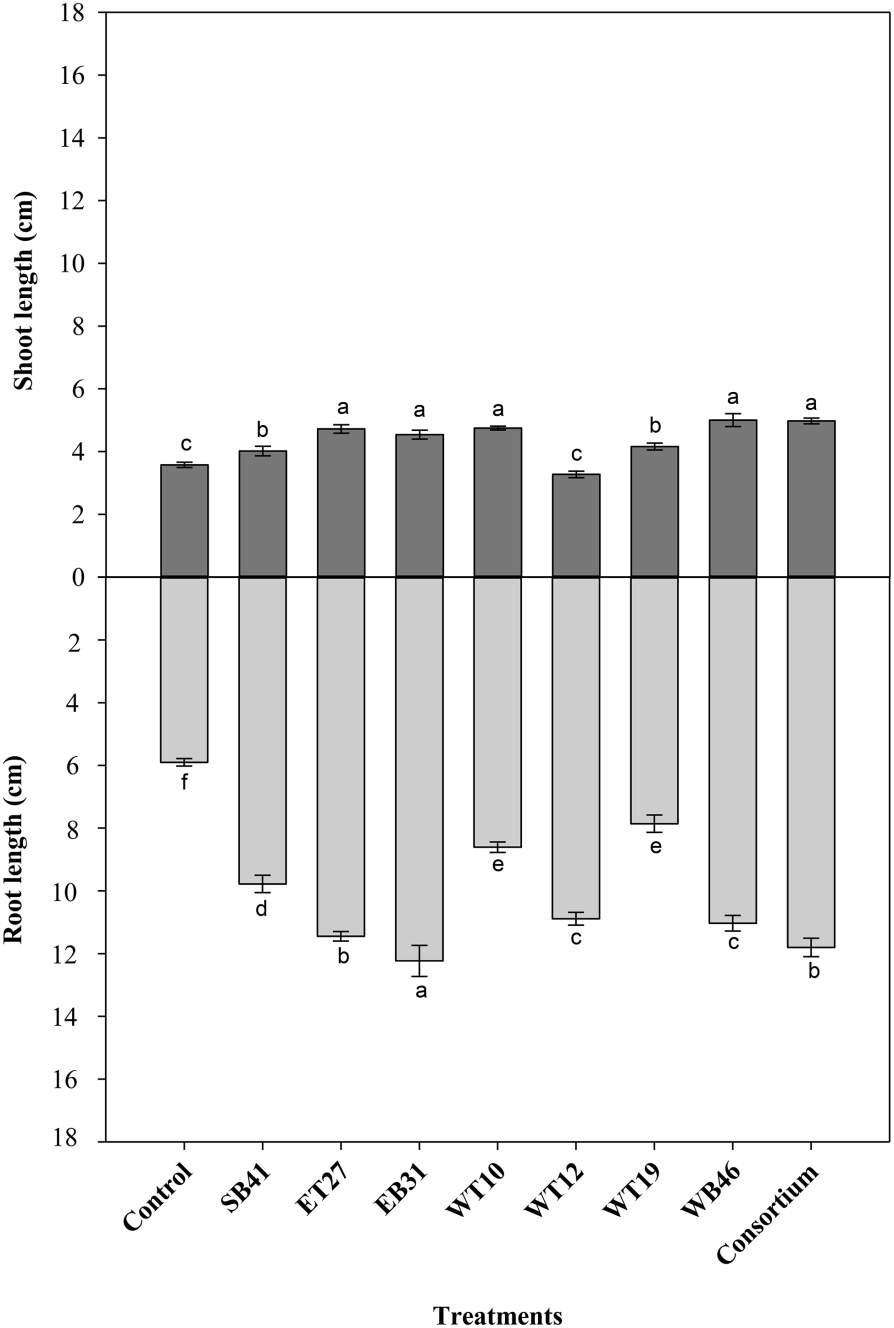
Effect of selected PGPR bacterial strains on root and shoot length (cm) of canola plants measured after 7 days of growth in the presence of 2% *n*-hexadecane. Error bars represent SDs, and different letters indicate significance according to Tukey’s *post-hoc* test at *p* ≤ 0.05.

**Figure 6:**
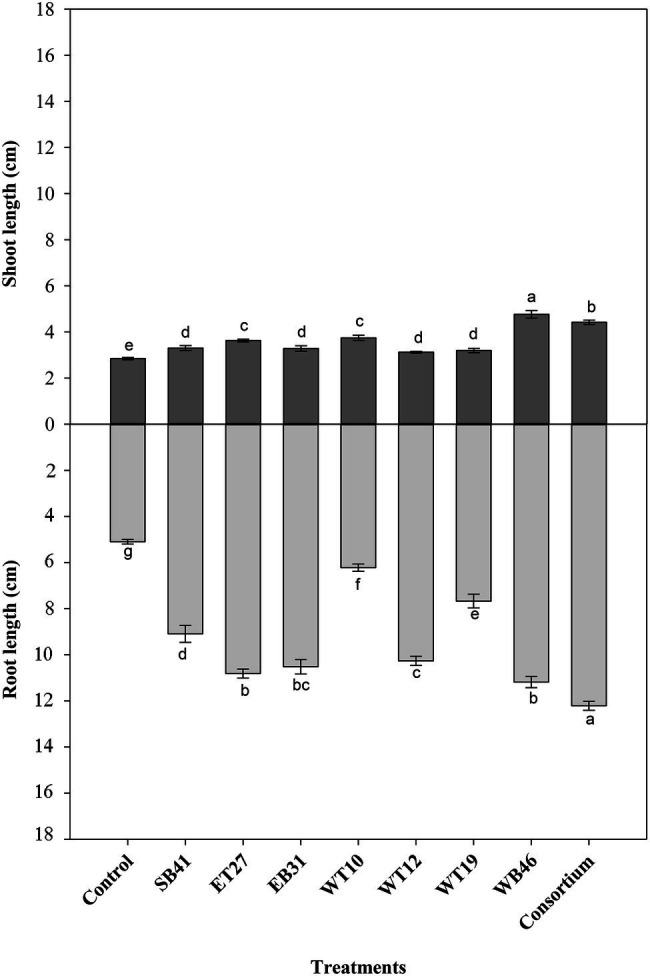
Effect of selected PGPR bacterial strains on root and shoot length (cm) of canola plants measured after 7 days of growth in the presence of 3% *n*-hexadecane. Error bars represent SDs, and different letters indicate significance according to Tukey’s *post-hoc* test at *p* ≤ 0.05.

When canola seedlings were grown in the presence of different concentrations of *n*-hexadecane (1%, 2%, and 3%), the resultant hydrocarbon stress caused a decrease in both root length and shoot length among most of the treatments (Figures 3–6). For example, canola seedlings treated with *S. pavanii* EB31, *G. amicalis* WT12, and *Pseudomonas plecoglossicida* ET27 grown in the presence of 3% *n*-hexadecane stress showed shoot lengths that were decreased by up to 60% compared to seedlings inoculated with the same strains grown in the absence of the hydrocarbon (Figures 3, 6). However, under 3% *n*-hexadecane amendment, all bacteria treatments significantly (*p* ≤ 0.05) increased their shoot length when compared with the control treatment (Figure 6). The highest shoot enhancement was induced by the isolate *Nocardia* sp. WB46 and the consortium treatment (Figure 6).

Unlike shoot length, canola seedlings grown under different concentrations of *n*-hexadecane showed an almost 16% decrease in root length when compared with seedlings grown in the absence of the hydrocarbon (Figures 3–6). In the presence of 3% *n*-hexadecane stress, all bacteria treatments significantly (*p* ≤ 0.05) increased root length when compared with the control treatment (Figures 4, 6). The highest root growth promotion was induced by the consortium treatment of *Nocardia* sp. WB46, *P. plecoglossicida* ET27, and *S. pavanii* EB31 (Figure 6).

### Growth of Bacterial Isolates in Different Concentrations of *n*-Hexadecane

**Figure 7:**
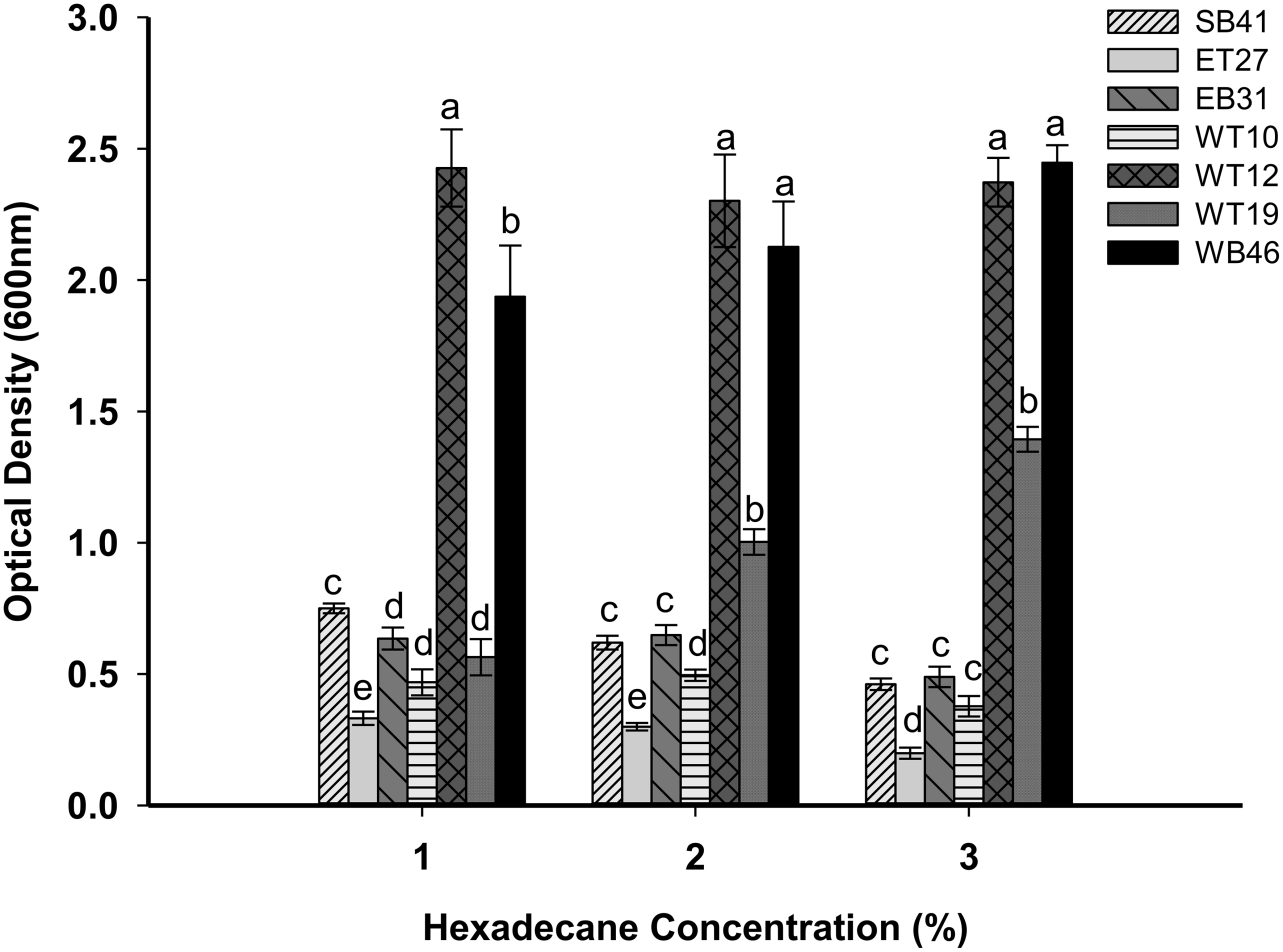
Effect of different concentrations of *n*-hexadecane (1%, 2%, and 3%) on bacterial growth of selected bacterial strains. All strains were grown for 7 days. Error bars represent SDs, and different letters indicate significance according to Tukey’s *post-hoc* test at *p* ≤ 0.05.

Bacterial isolates were grown in different concentrations of *n*-hexadecane (1%, 2%, and 3%) to determine the effect of increasing concentrations on bacterial growth. The results of this experiment indicated that when the concentration of *n*-hexadecane increased, the growth rate of some hexadecane-degrading bacteria was inhibited (Figure 7). For example, *B. megaterium* WT10 has an OD of 0.470 in 1% *n*-hexadecane, whereas this value decreased to 0.230 in the presence of the 3% concentration of *n*-hexadecane (Figure 7). Similar trends were observed for *plecoglossicida* ET27, *S. pavanii* EB31, and *Acinetobacter* sp. SB41. In contrast, *Nocardia* sp. WB46 and *Pseudarthrobacter siccitolerans* WT19 showed increased bacterial growth as the *n*-hexadecane concentrations increased (Figure 7). For example, *Nocardia* sp. WB46 had an OD of 1.6 with 1% *n*-hexadecane, whereas its growth rate increased to 2.3 with 3% *n*-hexadecane (Figure 7).

## Discussion

The use of plants in combination with bacteria possessing the ability to degrade PHCs and to promote plant growth is an efficient and environmentally sustainable strategy to remediate diesel-contaminated soils. High concentrations of PHCs can have phytotoxic effects on plants growing on contaminated soils (Baek et al., 2004). Therefore, the use of bacterial strains with multiple PGP and hydrocarbon degradation capabilities has crucial advantages for plants growing in such hostile environments.

To select bacterial isolates for the phytoremediation of diesel-contaminated soil, it is important to consider characteristics such as high degradation potential, the presence of alkane-degrading genes and a robust substrate affinity, as well as multiple PGP traits, such as the production of plant growth regulator substances and the ability to improve nutrient acquisition, which may enhance plant growth under contamination stress (Balseiro-Romero et al., 2017a).

We previously isolated and identified 438 PHC-degrading bacteria with multiple PGP characteristics from rhizosphere soil of *Salix purpurea* and *E. obtusa* plants growing in a highly PHC-contaminated site (Alotaibi et al., 2021b). In this study, we selected and characterized 50 bacterial isolates in depth based on their taxonomic and functional diversities and they were tested for their alkane degradation potential, PGP traits, and plant growth promotion potential under normal and stressed conditions using growth pouch assays.

PGPR regulate plant growth *via* diverse sets of mechanisms (Lugtenberg and Kamilova, 2009; Schlaeppi and Bulgarelli, 2015). In the present work, bacterial isolates exhibited several PGP traits, including P solubilization, IAA production, siderophore synthesis, ammonia production, ACCD activity, and N-fixation (Table 2). Ammonia production may play a role in enhancing plant growth through the accumulation of N and subsequently increasing biomass production (Marques et al., 2010). Ammonia production was the most common PGP trait observed among the strains. Similar results were reported in Dutta and Thakur (2017) in the characterization of 48 bacterial strains isolated from different tea cultivars in India. This suggest that ammonia production is among the mechanisms used by PGPR to stimulate plant growth. It has been shown that ammonia produced by PGPR supplies N to their host plants and thus promotes root and shoot elongation (Marques et al., 2010; Bhattacharyya et al., 2020).

The ability to fix nitrogen would provide a selective advantage for hydrocarbon-degrading bacteria used in phytoremediation applications, particularly in N-limited soils (Foght, 2018). Our results indicated that majority of diazotrophic bacteria belonged to *Gammaproteobacteria* (Table 2). In line with our findings, several reports indicate that diazotrophic bacteria predominate in PHCs-contaminated environments were affiliated to *Gammaproteobacteria* (Church et al., 2008; Radwan et al., 2010; Do Carmo et al., 2011) including taxa such as *Acinetobacter*, *Pseudomonas*, *Azotobacter*, *Stenotrophomonas*, and *Klebsiella* (Eckford et al., 2002; Dashti et al., 2009; Do Carmo et al., 2011; Foght, 2018; Alotaibi et al., 2021b). One of the constraint-limiting biodegradation activities of microbial communities in PHC-contaminated soils is the lack of sufficient nutrients especially nitrogen. Thus, application of diazotroph could offer a sustainable and efficient approach to enhance bioaugmentation and phytoremediation of PHC-contaminated soils (Dashti et al., 2009; Foght, 2018).

Bacteria capable of solubilizing inorganic forms of P may promote plant growth by improving the nutrient uptake of plants. The majority of bacterial isolates that showed P solubilization activity in this study belonged to *Proteobacteria* (Table 2). This corroborates previous reports about the ability of many bacterial strains isolated from plants growing in agricultural and contaminated soils and belonging to this phylum to have P solubilization abilities (Chowdhury et al., 2017; Pawlik et al., 2017; Lumactud and Fulthorpe, 2018). Iron-chelating siderophores are another important factor for PGP. PGPR produce siderophores that bind Fe^3+^ and render it available for reduction to Fe^+2^, a preferred form for plant roots uptake (Oleńska et al., 2020). Similar results were reported previously in Príncipe et al. (2007) in the characterization of PGPR isolates from saline soils. In a recent study, Eze et al. (2022) isolated a PGP and diesel-degrading bacterial consortium, dominated by *Alphaproteobacteria*, that led to a 66% increase in *Medicago sativa* biomass and resulted in 91% removal of diesel hydrocarbons in just 60 days. Functional metagenome analysis of the consortium revealed the presence of several genes responsible for PGP traits, including N-fixation, phosphate solubilization, and siderophore production (Eze et al., 2022). The prevalence of PGP genes in the consortium may account for not only the growth promotion of *M. sativa* but also their tolerance of diesel toxicity (Eze et al., 2022).

PGPR capable of lowering levels of ACC, a precursor of ethylene phyto-hormone, may stimulate growth and stress tolerance in plants under normal and stressed conditions (Glick, 2014). The high percentage of ACCD-producing bacteria in our study is in agreement with previous reports documenting the prevalence of this phenotype in various soil bacteria isolated from many stressed environments (Belimov et al., 2001; Mayak et al., 2004; Sandhya et al., 2010; Ali and Kim, 2018). Tara et al. (2014) reported that maximum bacterial population, plant biomass and hydrocarbon degradation activity were achieved for carpet grass plants growing in soil spiked with diesel and inoculated with bacterial strains (*Pseudomonas* sp. ITRH25, *Pantoea* sp. BTRH79, and *Burkholderia* sp. PsJN) possessing both alkane-degradation and ACCD activity, compared to bacterial strains possessing only alkane degradation activity.

IAA produced by PGPR is responsible for increasing root elongation, lateral root formation and root hairs, thus enhancing the water and nutrient uptake efficiency of the plant root system (Lugtenberg and Kamilova, 2009). Some recent studies have shown that rhizobacteria isolated from PHC-contaminated soil including strains of *Arthrobacter* sp., *Bacillus* sp., *Enterobacter* sp., *Rhodococcus* sp., *Pantoea* sp., *Pseudomonas* sp., *Stenotrophomonas* sp., and *Streptomyces* sp. are also PGPR-producing IAA (Pawlik et al., 2017; Balseiro-Romero et al., 2017a; Lumactud and Fulthorpe, 2018; Kidd et al., 2021). In a recent study, Li et al. (2021) reported a significant plant growth enchantment of ryegrass growing in PHC-contaminated soils and co-inoculated with bacterial strains *Arthrobacter pascen* and *Bacillus cereus*, possessing both IAA production and fluoranthene (Flu) degradation traits. Additionally, the Flu concentration was enhanced in the roots and shoots of inoculated plants (Li et al., 2021). The increase in the absorption and transport of Flu into plant tissues was attributed to the effect of IAA-producing bacteria on plants growth. IAA producing microbes would increase plant growth, which may increase the production of extra root exudates and lead to a higher transpiration rate, thus improving the rate of mineralization, solubility, and transport of Flu into the plant tissues (Técher et al., 2011; Li et al., 2021).

Plant-related factors in the rhizosphere, such as the production of organic compounds in root exudates, might affect the survival and colonization of PGPR and their ability to express many PGP activities (Drogue et al., 2012; Alemneh et al., 2021). Therefore, we used a plant-based strategy for screening PGPR regarding their PGP potential. In our study, numerous bacterial strains significantly increased the root elongation of canola plants (Figure 1). Our results are in line with the findings of Asghar et al. (2004), who screened the effect of 100 rhizobacterial strains on the promotion of canola root growth under gnotobiotic conditions and found that 58% enhanced root growth. Several studies have suggested that the PGPR isolates that most effectively promote plant growth produce both IAA and ACCD (Glick, 2014; Balseiro-Romero et al., 2017b; Kang et al., 2019). In our study, the highest root-growth promotion effect was observed with *Curtobacterium* sp. strain EA21. This strain produced the highest amount of IAA among all strains (44.13 μg ml^−1^; Table 2) and produced ACCD, as demonstrated by the plate assay and the positive PCR amplification result (Table 2). The cross-talk between IAA and ACCD is fundamental for PGPR to enhance root growth (Glick, 2014). Several studies have reported that PGPR producing IAA higher than 40 μg ml^−1^ inhibited root growth and seed germination (Pawlik et al., 2017; Alemneh et al., 2021) due to the stimulation of ethylene caused by the higher amount of IAA (Glick, 2014). However, if the bacterium has both IAA and ACC deaminase activities, then the ACCD would mediate the decreasing of ethylene production, thus permitting IAA synthesis, which could continue to enhance root growth (Glick, 2014; Kang et al., 2019; Alemneh et al., 2021). Several other PGPR in our study that promoted root growth and produced both IAA and ACC deaminase included strains such as *Microbacterium oxydans* strain SB39, *Pseudomonas mosselii* strain SB45, *Pseudomonas stutzeri* strain SB38, *P. plecoglossicida* strain ET27, *S. pavanii* strain EB31, *G. amicalis* strain WT12, and *Klebsiella oxytoca* strain EA5 (Figure 1). Interestingly, *B. megaterium* strain WT10, which was unable to express ACCD and IAA, significantly enhanced the root growth of canola plants compared to the control treatment (Figure 1). Therefore, the positive effect of this strain might be related to its ability to solubilize inorganic phosphate up to 690.86 μg ml^−1^ and its ammonia production (Table 2). Similar results were reported in Alemneh et al. (2021), who observed that several strains belonging to *Bacillus* spp. enhanced the growth and nodulation of chickpeas under gnotobiotic conditions. The improvement of plant growth was mainly related to the ability of these strains to express PGP traits other than IAA and ACCD (Alemneh et al., 2021). Interestingly, bacterial strains tested in this experiment showed growth-promoting potential for canola plants despite being isolated from different plant species. This fact suggests that these PGPR strains are non-host-specific, thus having huge potential as inoculants to promote plant growth in phytoremediation, as well as in organic agriculture.

Degradative bacteria can enhance the removal of alkanes and reduce the phytotoxicity of pollutants in soils due to their capability to possess hydrocarbon-degrading enzymes (van Beilen and Funhoff, 2007; Arslan et al., 2014). In our study, numerous bacterial strains had the potential to utilize aliphatic hydrocarbons (Table 3). Several authors have reported both large populations and high diversities of alkane-degrading bacteria in various habitats, ranging from marine environments to polar soils (Whyte et al., 2002; Yakimov et al., 2007; Jurelevicius et al., 2013; Lumactud et al., 2016; Pawlik et al., 2017).

Alkane hydroxylases (AHs) genes are responsible for the aerobic biodegradation of alkanes by bacteria (van Beilen and Funhoff, 2007). In our study, various bacterial strains harbored *AlkB* and *CYP153*-related AHs. These two AHs genes demonstrate a complementary substrate range. *AlkB* is involved in the degradation of medium-chain alkanes (C10-C20), whereas *CYP153* catalyzes the biodegradation of short-chain alkanes (C5-C16; Rojo, 2009; Ji et al., 2013; Wang and Shao, 2013). Similar results were reported by Pawlik et al. (2017), who screened 26 bacterial strains isolated from *Lotus corniculatus* and *Oenothera biennis* plants growing in a long-term polluted site, and found that 50% of these strains were equipped with *CYP153* genes.

Previous research has shown that AH genes are often associated with *Betaproteobacteria* and *Gammaproteobacteria*, particularly the *Pseudomonas* genus (van Beilen and Funhoff, 2007; Liu et al., 2014; Garrido-Sanz et al., 2019; Eze et al., 2021). In our study, AH-degrading genes were also found in strains that belonged to *Betaproteobacteria* and *Gammaproteobacteria*; in addition, members of the *Actinobacteria*, such as *Gordonia*, *Arthrobacter*, *Nocardia*, *Rhodococcus*, and *Rhodococcus*, were found to harbor these genes (Table 3). The wider taxonomic affiliations of bacterial strains capable of metabolizing alkanes demonstrate the potential of this culture collection for the remediation of diesel-contaminated soils.

Interestingly, several strains tested in this study had multiple *AlkB* and *CYP153* genes coexisting together (Table 3). The co-occurrence of multiple AHs has been reported previously in several bacterial strains, such as *Acinetobacter* sp. ADP1 (Barbe et al., 2004), *Dietzia* sp. DQ12-45-1b (Nie et al., 2011), and *Amycolicicoccus subflavus* DQS3-9A1 (Nie et al., 2014b). Undoubtedly, the coexistence of multiple AH genes in one bacterium would extend the alkane substrate range, thus enhancing the adaptation ability and subsequently the degradation potential of the host bacterium (Sun et al., 2018).

Under contaminant stress conditions (the second growth pouch experiments with various *n*-hexadecane concentrations), the inoculation of canola seeds with the selected PGPR strains either alone or in consortia generally provoked a significant increase in both the root length and shoot length of canola seedlings when compared with control plants (Figures 3–6). This indicates that PGPR inoculants exert a positive effect on plant growth under such stressful conditions. In agreement with our results, Balseiro-Romero et al. (2017b) reported that the inoculation of *Cytisus striatus* L. and *Lupinus luteus* L plants, grown in 1.25% diesel-contaminated soil in a pot experiment, with diesel-degrading bacterial strains with multiple PGP activities significantly improved plant growth. In the present experiment, selected hexadecane-degrading strains were evaluated for their ability to promote the growth of canola plants under increasing hexadecane concentrations (Table 3). Additionally, these selected hexadecane-degrading strains possessed multiple PGP traits (Table 2). Among the hexadecane-degrading strains, after the consortium treatments, the actinobacterium *Nocardia* sp. strain WB46 was found to be the best plant growth promoter among all the strains assessed (Figure 6). This strain showed robust growth on hexadecane as a sole energy source (Figure 7). Genome analyses revealed that *Nocardia* sp. strain WB46 contains three copies of the *alkB* gene (unpublished data). *AlkB* is a class of alkane hydroxylase enzymes that is responsible for the microbial degradation of oil and fuel additives, as well as many other compounds (van Beilen and Funhoff, 2007; Nie et al., 2014a). *Nocardia* sp. strain WB46 was also shown to possess several PGP activities, such as IAA, siderophore, and ammonia production (Table 2). IAA is a phytohormone responsible for increasing root elongation and the formation of lateral root and root hairs, thus enhancing the water and nutrient uptake efficiency of plant root systems (Lugtenberg and Kamilova, 2009), whereas the production of siderophores and ammonia plays a role in enhancing plant growth by increasing the nutrient acquisition efficiency of Fe^+2^ and N, respectively (Lugtenberg and Kamilova, 2009; Marques et al., 2010).

Other hexadecane-degrading isolates, specifically *P. plecoglossicida* ET27 and *S. pavanii* EB31, exhibited excellent plant growth promotion potential (Figure 6). Although these two isolates do not utilize hexadecane as efficiently as *Nocardia* sp. WB46 (Figure 7), they were shown to possess strong PGP capabilities (Table 2). *Pseudomonas plecoglossicida* ET27 was able to produce all PGP traits under investigation in this study. In agreement with our results, Balseiro-Romero et al. (2017b) reported the characterization of *Pseudomonas* strain 12, which was isolated from the rhizosphere of poplar plants growing in a diesel-contaminated site. In their study, *Pseudomonas* stain 12 was able to solubilize P, produce siderophore, synthesize IAA and produce ACCD, as well as promoting plant growth when used as an inoculum to enhance the growth of plants growing in diesel-contaminated soils (Balseiro-Romero et al., 2017b). *Stenotrophomonas pavanii* EB31 was also shown to possess all PGP features. Several recent studies highlighted the potential of the members of genus *Stenotrophomonas* having multiple PGP traits to be used as inoculants in the bioremediation of PHC-contaminated soils (Pawlik et al., 2017; Lumactud and Fulthorpe, 2018; Mitter et al., 2019; Alotaibi et al., 2021b).

Interestingly, *P. plecoglossicida* ET27 and *S. pavanii* EB31 contain genes for N-fixation and alkane degradation. Earlier studies reported that other N-fixers such as *Frankia* spp. were found to harbored alkane genes in addition *nifH* gene (Rehan et al., 2016). Diazotroph capable of coupling N-fixation to hydrocarbon degradation represent a key strategy to promote plant growth in N-limited marginal lands such as PHC-contaminated soils (Foght, 2018). Thus, enhancing the efficiency of phytoremediation of PHC-contaminated soils.

Our results along with previous reports (Tara et al., 2014; Balseiro-Romero et al., 2017a,b; Kidd et al., 2021) support our hypothesis that bacteria with multiple PGP and pollutant degradation characteristics performed better than strains with only one of these traits. In addition, hexadecane-degrading activity could be considered itself a PGP feature, because pollutants have a harmful effect on plant growth and development. Given the potential for positively influencing plant growth, exploiting these bacteria is a major goal for environmental and agricultural biotechnologies (Quiza et al., 2015). Such an achievement could enhance our ability to promote efficient soil nutrient use to increase plant growth, while reducing the toxicity of PHC pollutants. Our study clearly demonstrated that some bacterial taxa exhibit traits of PGP and capacity of degradation of PH pollutants. Integration of bacterial taxa that performed better for each test, can help to develop different formulations of bioinoculants. Further compatibility and formulation tests of these bioinoculants must avoid competition between isolates and allow the maintenance of microbial propagule survival for long periods of time and assure their ability to improve the growth of plants under various field conditions.

## Conclusion

In conclusion, the results of our study suggest that the screening of rhizobacteria for *in vitro* PGP activities, aliphatic hydrocarbon degradation potential, and root growth promotion under gnotobiotic conditions is an effective approach for the selection of efficient PGPR candidates for bioremediation biotechnology applications. After several rounds of screenings, bacterial strains *Nocardia* sp. WB46, *P. plecoglossicida* ET27 and *S. pavanii* EB31 showed the highest growth stimulation when grown under the presence of 3% *n*-hexadecane. These isolates originated from a unique site with high concentration of PHC pollution scored positive for PGP traits and hexadecane degradation potentials, indicating the potential to serve as inoculants for assisting the phytoremediation of diesel-contaminated soils. Additionally, with this culture collection in hand, a better understanding of the role of plant growth promotion in the phytoremediation of PHC-contaminated soils can be achieved through additional phenotypic and in planta characterization, whole genome sequencing, and the construction of bacterial consortia for field applications.

## Data Availability Statement

The datasets presented in this study can be found in online repositories. The names of the repository/repositories and accession number(s) can be found in the article/Table 1.

## Author Contributions

FA performed experiments and experimental design, analyzed data, and wrote the manuscript draft. MS-A contributed to conceptualization, experimental design, co-supervision, and manuscript editing. MH contributed to concepts, design, supervision, and manuscript writing. All authors contributed to the article and approved the submitted version.

## Funding

This study was supported by funds from Natural Sciences and Engineering Research Council of Canada (NSERC) discovery grant to MH (grant number: RGPIN-2018-04178), Genome Quebec, Genome Canada, FA was also supported by a grant from King Saud University *via* the Saudi Arabian Cultural Bureau in Ottawa which are gratefully acknowledged.

## Conflict of Interest

The authors declare that the research was conducted in the absence of any commercial or financial relationships that could be construed as a potential conflict of interest.

## Publisher’s Note

All claims expressed in this article are solely those of the authors and do not necessarily represent those of their affiliated organizations, or those of the publisher, the editors and the reviewers. Any product that may be evaluated in this article, or claim that may be made by its manufacturer, is not guaranteed or endorsed by the publisher.

## Supplementary Material

The Supplementary Material for this article can be found online at: https://www.frontiersin.org/articles/10.3389/fmicb. 2022.863702/full#supplementary-material
