# Supplementary material for: In-Depth Characterization of Plant Growth Promotion Potentials of Selected Alkanes-Degrading Plant Growth-Promoting Bacterial Isolates
Source: Front Microbiol. 2022 Mar 29;13:863702. doi: 10.3389/fmicb.2022.863702 (PMC9002309; doi:10.3389/fmicb.2022.863702)
Supplement: Supplementary file 1 [file Table_1.DOCX]

**Table S1** PCR conditions used to amplify hydrocarbon degradation genes and plant growth promotion-related genes present in bacterial genomes.

| Target gene | Primers | PCR Conditions | Reference |
| --- | --- | --- | --- |
| Alkane monooxygenase (*alkB*) | AlkB-F  AlkB-R | Initial denaturation step of 4 min at 94 °C; 32 cycles of 30 s at 94 °C, 30 s at 55 °C, and 1 min at 72 °C and a final elongation step of 10 min at 72 °C. | Kloos et al., 2006 |
| Cytochrome P450 hydroxylase (*CYP153*) | P450-F  P450-R | Initial denaturation step of 4 min at 94 °C; 32 cycles of 30 s at 94 °C, 30 s at 52 °C, and 1 min at 72 °C and a final extension step of 10 min at 72 °C. | Wang et al., 2010 |
| Naphthalene dioxygenase (*nah*) | nah-F  nah-F | Initial denaturation step of 5 min at 95 °C; 30 cycles of 1 min at 95 °C, 1 min at 47 °C, and 2 min at 72 °C and a final elongation for 10 min at 72 °C. | Baldwin et al., 2003 |
| ACCD enzyme (*acdS*) | F1936  F1938 | Initial denaturation step of 5 min at 95 °C; 35 cycles of 30 s at 95 °C, 30 s at 50 °C, and 30 s at 72 °C and a final elongation step of 7 min at 72 °C. | Blaha et al., 2006 |
| Nitrogen fixation (*nifH*) | nifH-F  nifH-R | Initial denaturation step of 5 min at 95 °C; 1 cycle of  20 s at 96 °C, 30 s at 65 °C, and 30 s at 72 °C; 2 cycles of 20 s at 96 °C, 30 s at 62 °C, and 35 s at 72 °C; 3 cycles of 20 s at 96 °C, 30 s at 59 °C, and 40 s at 72 °C; 4 cycles of 20 s at 96 °C, 30 s at 56 °C, and 45 s at 72 °C; 5 cycles of  20 s at 96 °C, 30 s at 53 °C, and 50 s at 72 °C; 25 cycles of 20 s at 94 °C, 45 s at 50 °C, and 60 s at 72 °C; and an extension step of 10 min at 72 °C. | Rösch et al., 2002 |
